# Cancer and mTOR inhibitors in kidney transplantation recipients

**DOI:** 10.7717/peerj.5864

**Published:** 2018-11-08

**Authors:** Chih-Chin Kao, Jia-Sin Liu, Yu-Kang Chang, Ming-Huang Lin, Yen-Chung Lin, Hsi-Hsien Chen, Wei-Chiao Chang, Chih-Cheng Hsu, Mai-Szu Wu

**Affiliations:** 1 Graduate Institute of Clinical Medicine, College of Medicine, Taipei Medical University, Taipei, Taiwan; 2 Division of Nephrology, Department of Internal Medicine, Taipei Medical University Hospital, Taipei, Taiwan; 3 Department of Internal Medicine, School of Medicine, Taipei Medical University, Taipei, Taiwan; 4 Division of Geriatrics and Gerontology, Institute of Population Health Sciences, National Health Research Institutes, Zhunan, Taiwan; 5 Department of Medical Research, Tungs’ Taichung Metroharbor Hospital, Wuchi, Taichung, Taiwan; 6 Department of Clinical Pharmacy, College of Medicine, Taipei Medical University, Taipei, Taiwan; 7 Master Program for Clinical Pharmacogenomics and Pharmacoproteomics, School of Pharmacy, Taipei Medical University, Taipei, Taiwan; 8 Department of Health Services Administration, China Medical University, Taichung, Taiwan; 9 Division of Nephrology, Department of Internal Medicine, Shuang-Ho Hospital, New Taipei City, Taiwan

**Keywords:** Chronic kidney disease, Cancer, Kidney transplantation, Mortality, mTOR inhibitors

## Abstract

**Background:**

Previous studies show that mTOR inhibitors decrease the risk of cancer development after kidney transplantation. However, the effect of cumulative doses of mTOR inhibitors on cancer after kidney transplantation is not well known.

**Methods:**

In the current study, patients were registered into a national database in Taiwan. Between year 2000 and 2013, 4,563 patients received kidney transplantation. They were divided into two groups, according to mTOR inhibitors usage. The cumulative dose of mTOR inhibitors was recorded. Patients were followed-up until de novo cancer development, death, or the end of 2014.

**Results:**

Patients were divided into two groups: mTOR inhibitors users (study group, *n* = 828) and mTOR inhibitors non-users (control group, *n* = 3,735). The median follow-up duration was 7.8 years. The risk of de novo cancer (hazards ratio (HR) 0.80, 95% CI [0.60–1.09], *p* = 0.16) and risk of death (HR 1.14, 95% CI [0.82–1.60], *p* = 0.43) was not different between mTOR inhibitor user and non-user groups. Neither high- nor low-dose exposure to mTOR inhibitors was associated with increased risk of cancer or mortality. Analysis of cancer subtypes showed no influence by mTOR inhibitors. In addition, the cause of mortality was not significantly different between the two groups.

**Discussion:**

We could not find the association of mTOR inhibitors use and risk of de novo cancer development or mortality in patients with kidney transplantation in Chinese patients. Cumulative exposure to mTOR inhibitors did not change the results.

## Introduction

Over 75% of living kidney recipients survived more than 10 years (USRDS, 2015). Cancer development due to long term exposure to immunosuppressives has become a growing problem leading to poor prognosis in these patients (Briggs, 2001). The cancer incidence is three to five times higher in these patients as compared to that in the general population (Birkeland, Lokkegaard & Storm, 2000). Several causative factors are associated with increased risk of cancer after kidney transplantation. The total burden of immunosuppressives rather than any single agent may account for the increased risk of malignancy (Morath et al., 2004). mTOR inhibitors emerged in 1999 (Kahan, 2000; Kahan et al., 1999) and were identified as protective agents against cancer (Faivre, Kroemer & Raymond, 2006; Wander, Hennessy & Slingerland, 2011).

mTOR is a protein kinase belonging to the phosphatidylinositol 3-kinase (PI3K/Akt) pathway, which controls cell survival, angiogenesis, and proliferation. This protective effect derives from the success of mTOR inhibitors in advanced renal cell carcinoma (RCC). RCC is characterized by a persistent activation of hypoxia inducible factor-1 α (HIF-1α) due to a mutation in the Von Hippel–Lindau gene (Baldewijns et al., 2010). HIF-1α is regulated by mTOR, therefore mTOR inhibitors may down-regulate HIF-1α, reducing the burden of RCC. In addition, a mutation in the *PTEN* gene may lead to over-activation of the PI3K/Akt/mTOR pathway. This gene mutation has been observed in a variety of malignancies (Chalhoub & Baker, 2009).

The predominant type of cancer that develops after kidney transplantation differs from western to eastern countries. A US Cincinnati registry showed that the most frequent types of cancer occurrences after kidney transplantation are lymphoproliferative disorder and squamous cell carcinoma (Zeier et al., 2002). A UK registry reported a higher risk of lip cancer, non-melanoma skin cancer, Kaposi sarcoma, and non-Hodgkin’s lymphoma (Collett et al., 2010). In contrast, several studies reported renal, bladder, and liver cancers as the most frequently observed cancers in eastern countries (Li et al., 2012; Zhang et al., 2014). Cheung et al. (2012) showed that non-Hodgkin lymphoma, kidney, and bladder cancer were the leading cancer subtypes in Hong Kong. The studies on the cancer-protective effect of mTOR inhibitors were mainly conducted in western countries. Few data has been reported on the anti-cancer effects and overall outcomes of mTOR inhibitors in Asian populations. Differences in cancer epidemiology and immunosuppressive treatment may result in a different outcome in these populations. In addition, to the best of our knowledge, most of the previous studies focus on conversion from calcineurin inhibitors (CNIs) to mTOR inhibitors and their protective effects against skin cancer (Campbell et al., 2012; Euvrard et al., 2012). A German study showed that the use of *de novo* mTOR inhibitors is associated with a lower risk of skin basal cell carcinoma (Opelz et al., 2016). No previous studies have investigated the effect of cumulative doses of mTOR inhibitors on cancer risk after kidney transplantation. We aimed to study the effect of mTOR inhibitors on *de novo* cancer development, and all-cause mortality after kidney transplantation, using a nation-wide database.

## Materials and Methods

### Data source

We collected the data of interest from the National Health Insurance Research Database (NHIRD), which was established by the Taiwan Bureau of National Health Insurance (TBNHI), starting in 1995 and covering over 99% of residents in Taiwan (Cheng, 2003). It is a national and comprehensive database, including information on sex, age, drug prescription, medical procedures, and diagnosis code using the International Classification of Diseases 9th revision—Clinical Modification (ICD-9-CM). This database is available for research and encrypted to prevent the identification of subjects. Therefore, this study was waived from a full review by the Institutional Review Board of Taipei Medical University (N201702024).

### Study design

Our aim was to investigate the potential association of mTOR inhibitors with the risk of *de novo* cancer development and all-cause mortality. All enrolled patients were 20 years of age or older and received kidney transplantation between January 1st, 2000 and December 31st, 2013. The patients with a diagnosis code of (ICD-9-CM code V42.0 and 996.81) in the catastrophic illness database registry were included. For patients who received surgery in Taiwan, the index date was defined by the kidney transplantation surgery code (76020A, 76020B, 97416A, 97416K, 97417B); for patients who received surgery overseas, their index date was the first date of prescription of immunosuppressives with the diagnosis code of kidney transplantation. We excluded patients who had all of the following criteria:

- malignancy history before kidney transplantation
- those for whom age or gender was unknown
- whose steroid usage was for less than 30 days within 1 year after kidney transplantation
- whose follow-up duration was less than 1 year or who were dead

Based on the drug prescription data, those patients who adhered to mTOR inhibitor treatment (either sirolimus or everolimus) ≧180 cumulative days within the first year of kidney transplantation were included in the study group (mTOR inhibitors users) to determine its contributory risk of cancer; The other patients were included in the control group (mTOR inhibitors non-users).

We also added demographic variables, including age, gender, dialysis vintage, diagnostic codes, concomitant drug prescriptions, and hospital type (medical center, regional hospital, and district hospital). The potentially selective confounding drugs included angiotensin-converting enzyme inhibitors/angiotensin II receptor blockers, β-blockers, aspirin, non-steroid anti-inflammatory drugs, statins, cyclosporine, tacrolimus, mycophenolic acid (MPA), and antithymocyte globulin (ATG).

### Exposure to mTOR inhibitors

The anatomical therapeutic chemical classification system codes were used to identify mTOR inhibitors. The defined daily dose (DDD) was defined as the average maintenance dose per day of a drug used for its main indication in adults, as recommended by the World Health Organization (Kahan et al., 1999). Sirolimus three mg and everolimus 1.5 mg are equal to 1DDD. The drug quantity, dose, and prescription date were collected from the database. We assumed dose dependent effect of mTOR inhibitors could be determined by the cumulative dose of mTOR inhibitors. The cumulative DDD (cDDD) was defined as the additive sum of DDD for mTOR inhibitors during the study period. The average daily DDD was calculated as cDDD divided by the total prescription days. The exposure of mTOR inhibitors was determined by the amount of cDDD and the average daily DDD. The patients with single mTOR inhibitor use (either sirolimus only or everolimus only) were analyzed. As a result, patients were divided into three tertiles. To test the effect of exposure on the patients’ outcomes, the three tertiles of mTOR inhibitor users were compared to that of non-mTOR inhibitor users.

### Outcomes

The patients were followed-up until *de novo* cancer development, death, or the end of 2014. Our primary outcomes were either *de novo* cancer development or all-cause mortality. Cancer development was defined by its diagnosis code (ICD-9-CM 140-208.91) on the hospitalized discharge diagnosis. The specific cancer subtype was also investigated. Patients who develop more than two types of cancer were classified as “many cancers.” In addition, to identify the specific cause of death, we used the NHIRD database that registers medical certification of death profiles in Taiwan. The “leading” cause of death was defined as the cause of mortality and further divided into cardiovascular (CV), a composite of CV and renal, infectious, malignancy, and others.

### Statistical analysis

SAS version 9.4 was used for statistical analysis. Chi-square test or *t*-test were performed for demographics comparison as indicated. The Kaplan–Meier method was applied to calculate the cumulative incidence of *de novo* cancer development and all-cause mortality between mTOR inhibitors users and mTOR inhibitors non-users groups. We conducted a multivariate Cox proportional hazards model analysis after adjustment for confounders. Covariates such as age, gender, dialysis vintage, transplant domestic/overseas, comorbidities, and medications were included in the model. Use of mTOR inhibitors was treated as a time-dependent variable to prevent immortal time bias (Suissa, 2008) which meant patients were considered as mTOR inhibitors non-users until initiating mTOR inhibitors. We applied this time-dependent model as the main model. *p* < 0.05 was considered a threshold for statistical significance. Bonferroni correction with multiple test adjustment was performed to control the familywise error rate (McDonald, 2014).

To alleviate the effects of skewness in the two groups, minimize the effects of different baseline demographics on the outcome and avoid the confounding indications, we applied a 1:1 propensity score matching model. The propensity score in each patient was calculated according to a predictive method of mTOR inhibitors users or non-users by multivariate logistic regression. To assess the robustness of the results, we further analyzed the outcomes in stratified subgroups and drew a forest plot for discrimination. To minimize the potential bias of 180-day prescriptions on mTOR inhibitors users, we conducted a sensitivity analysis by setting a cumulative “30-day” or “90-day” range for the mTOR inhibitors users, and by setting patients without any mTOR inhibitors use as a control group.

## Results

### Characteristics of the study population

**Figure 1:**
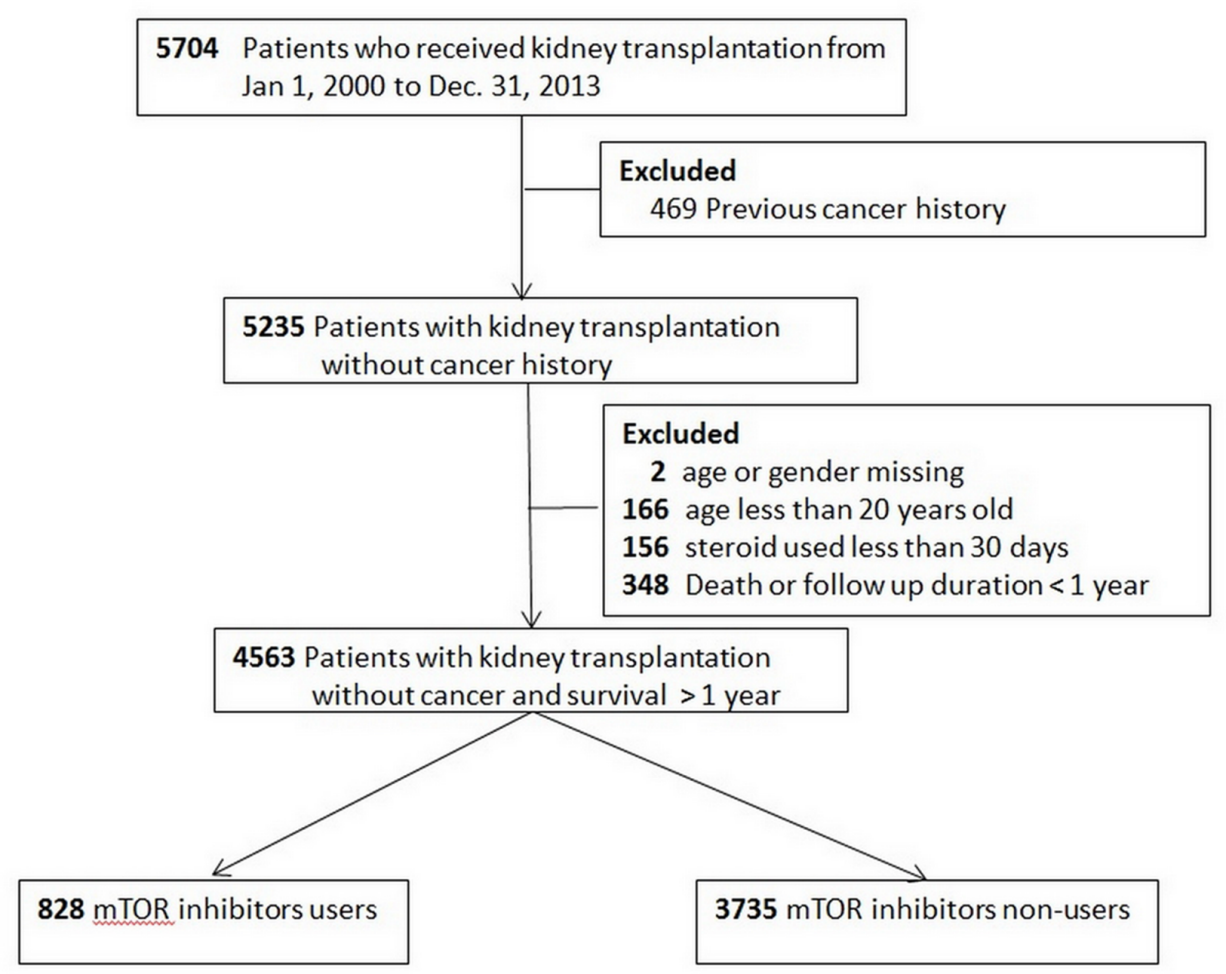
Flow chart of patient selection.

The flow chart of patient selection is illustrated in Fig. 1. A total of 828 patients were mTOR inhibitors users (study group) and 3,735 were mTOR inhibitors non-users (control group). The demographics and the accompanying prescribed medications are shown in Table 1. The study group was younger, involved more male participants, and characterized by the fact that most patients underwent transplantation in Taiwan. The study group was more likely to receive β-blocker, statins, tacrolimus, and ATG; whereas cyclosporin, and MPA were more likely administered in the control group.

**Table 1:** Demographics of kidney transplantation recipients according to mTOR inhibitor treatment.

	Number of mTOR inhibitor users (*n* = 828)	Number of mTOR inhibitor non-users (*n* = 3,735)	*p*-value
Mean age	44.8 ± 11.5	47.0 ± 11.0	<0.001
Age			<0.001
20–39	268 (32.4%)	942 (25.2%)	
40–54	378 (45.7%)	1,813 (48.5%)	
55–65	160 (19.3%)	806 (21.6%)	
>65	22 (2.7%)	174 (4.7%)	
Gender			0.010
Man	477 (57.6%)	1,967 (52.7%)	
Woman	351 (42.4%)	1,768 (47.3%)	
Dialysis vintage	3.6 ± 3.0	3.1 ± 3.0	0.16
Transplant			<0.001
Domestic	641 (77.4%)	2,034 (54.5%)	
Overseas	187 (22.6%)	1,701 (45.5%)	
Comorbidity			
DM	114 (13.8%)	557 (14.9%)	0.40
HTN	519 (62.7%)	2,232 (59.8%)	0.12
CHF	58 (7.0%)	227 (6.1%)	0.32
Drug used			
ACEI / ARB	255 (30.8%)	1,140 (30.5%)	0.877
β-blocker	427 (51.6%)	1,642 (44.0%)	<0.001
NSAID+Aspirin	154 (18.6%)	859 (23.0%)	0.006
Statins	312 (37.7%)	953 (25.5%)	<0.001
Cyclosporin	292 (35.3%)	1,505 (40.3%)	0.007
Tacrolimus	656 (79.2%)	2,669 (71.5%)	<0.001
Mycophenolic acid	529 (63.9%)	2,916 (78.1%)	<0.001
ATG	7 (0.8%)	25 (0.7%)	0.02

**Note:**

ACEI, angiotensin-converting enzyme inhibitors; ARB, angiotensin II receptor antagonist; ATG, antithymocyte globulin; CHF, congestive heart failure; DM, diabetes mellitus; HTN, hypertension; NSAID, non-steroidal anti-inflammation drugs.

### Outcomes

**Figure 2:**
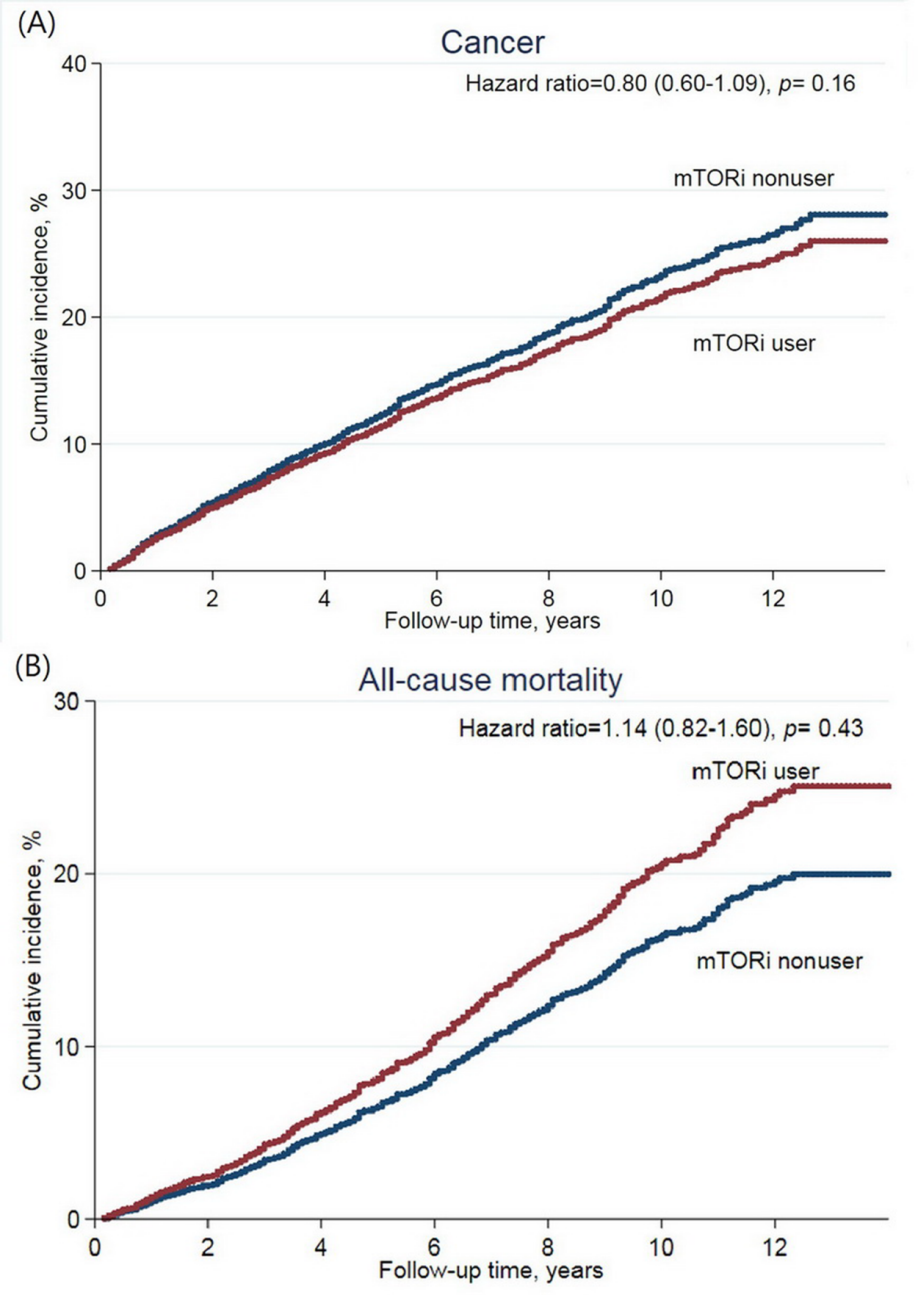
Cumulative incidence of (A) de novo cancer development, and (B) all-cause mortality after kidney transplantation according to mTOR inhibitor treatment.

The median follow-up was 7.8 years. There were a total of 87 (11%) patients that developed *de novo* cancer in the study group and a corresponding 628 (16.8%) patients in the control group. Incidence rate (IR) was 17.0 in the study group and 23.6 in the control group per 1,000 person-years (Table 2). The risk of cancer development was not reduced by mTOR inhibitors (adjusted hazards ratio (HR) 0.80 (95% CI [0.60–1.09], *p* = 0.16)) (Fig. 2A). The most observed cancer types consisted of kidney, bladder, and liver cancer. In specific cancer analysis, mTOR inhibitors use was not associated with the protective effect on a specific cancer. Similar results were found in the propensity score matching model (Table S1).

**Table 2:** Incidence and risk of *de novo* cancer development after kidney transplantation according to mTOR inhibitor treatment in time-dependent model.

Outcome	With mTORi		Without mTORi		Adjusted HR* (95% CI)	*p*-value
	Events	IR	Events	IR		
Any cancer	87	17.0	628	23.6	0.80 (0.60–1.09)	0.16
Kidney	17	3.1	109	3.8	1.29 (0.62–2.66)	0.50
Bladder	17	3.2	152	5.3	0.53 (0.28–1.00)	0.05
Lung	3	0.6	26	0.9	0.65 (0.14–3.11)	0.59
Liver	6	1.1	65	2.2	0.84 (0.28–2.56)	0.76
Colorectal	4	0.7	27	0.9	1.73 (0.39–7.66)	0.47
Breast	1	0.2	23	0.8	0.29 (0.03–3.27)	0.32
Hematological	5	0.9	24	0.8	2.03 (0.46–8.92)	0.35
Skin	1	0.2	4	0.1	5.66 (0.14–226)	0.36
Prostate	3	0.6	17	0.6	1.26 (0.21–7.49)	0.80
Others	30	5.6	208	7.3	0.73 (0.44–1.21)	0.23
Many cancers(≧2)	3	0.6	35	1.3	1.12 (0.22–5.75)	0.89

**Notes:**

IR: incidence rate, per 1,000 person-years.

* Adjusted for age, gender, dialysis vintage, transplant within/overseas, DM, HTN, CHF, ACEi/ARB, Beta-blocker, Aspirin, NSAID, Statins, Cyclosporin, Tacrolimus, Mycophenolic acid, ATG.

Regarding all-cause mortality, 79 patients (9.5%) of the study group and 461 patients (12.3%) of the control group died during the study period (IR: 14.5 vs. 15.7 per 1,000 person-years, respectively) (Table 3). The risk of all-cause mortality was not increased in the study group (adjusted HR 1.14 (95% CI [0.82–1.60], *p* = 0.43)) (Fig. 2B). Malignancy-related mortality accounted for 29.1% of all-cause mortality in the study group and for 30.6% in the control group. The composite outcome of CV and renal-related death accounted for 34.1% in the study group and 34.3% in the control group. mTOR inhibitors were not associated with an increased risk in any specific cause of mortality. In the propensity score matching model, the study group was associated with reduced risk of the composite outcome (Table S2).

**Table 3:** Incidence and risk of all-cause mortality after kidney transplantation according to mTOR inhibitor treatment in time-dependent model.

Outcome	With mTORi		Without mTORi		Adjusted HR* (95% CI)	*p*-value
	Events	IR	Events	IR		
All-cause mortality	79	14.5	461	15.7	1.14 (0.82–1.60)	0.43
Malignancy	23	4.2	141	4.8	1.26 (0.67–2.35)	0.47
CV	9	1.6	52	1.8	1.14 (0.42–3.12)	0.79
CV/Renal	27	4.9	158	5.4	0.95 (0.54–1.67)	0.87
Infectious	6	1.1	36	1.2	1.42 (0.42–4.83)	0.57
Accident	1	0.2	7	0.2	0.31 (0.03–3.84)	0.36
Others	22	4.0	119	4.1	1.41 (0.73–2.69)	0.30

**Notes:**

CV, cardiovascular; IR, incidence rate, per 1,000 person-years.

* Adjusted for age, gender, dialysis vintage, transplant within/overseas, DM, HTN, CHF, ACEi/ARB, Beta-blocker, Aspirin, NSAID, Statins, Cyclosporin, Tacrolimus, Mycophenolic acid, ATG.

**Figure 3:**
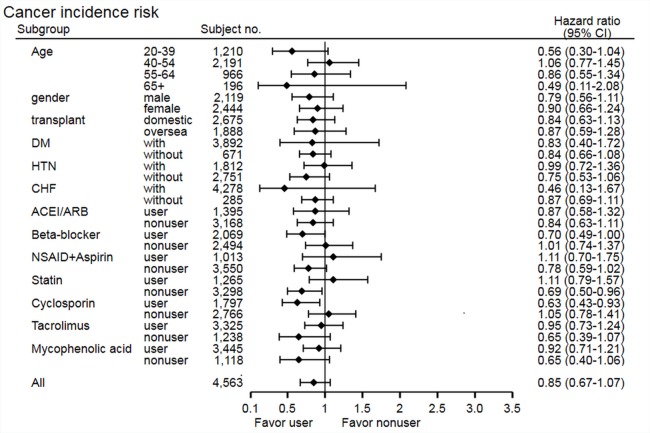
Subgroup analysis of risk of cancer development.

**Figure 4:**
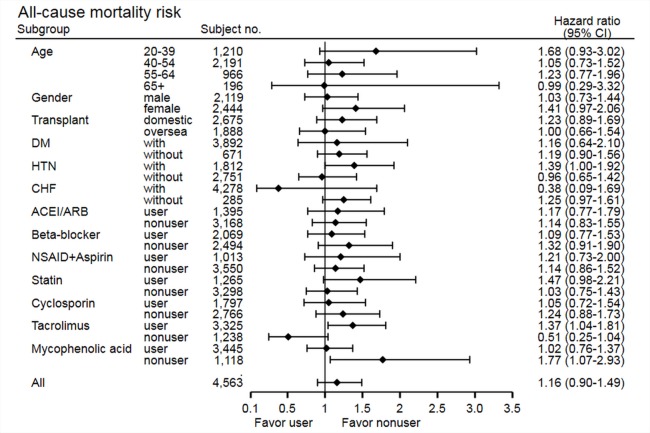
Subgroup analysis of risk of all-cause mortality.

The stratified analysis of risk of cancer development showed non-significant findings across most subgroups, except in the statin non-user group (HR 0.69, 95% CI [0.50–0.96], *p* = 0.03) and cyclosporine user group (HR 0.63, 95% CI [0.43–0.93], *p* = 0.02) (Fig. 3). In the statin non-user group, the cancer risk reduction mainly came from the protective effect on bladder cancer development (HR 0.38, 95% CI [0.15–0.95], *p* = 0.03). The stratified analysis of all-cause mortality showed similar findings, but study group patients have higher risk of mortality in the tacrolimus user group (HR 1.37, 95% CI [1.04–1.81], *p* = 0.02) and MPA non-user group (HR 1.77, 95% CI [1.07–2.93], *p* = 0.03) (Fig. 4). Further analysis showed no significantly increased risk of specific cause of mortality in the two subgroups (data not shown), and these subgroup findings were not significant after Bonferroni correction (Tables S3 and S4).

In the sensitivity analysis, we applied study group patients as cumulative “30-day” or “90-day” of mTOR inhibitors use, and the results were not changed. The patients without any mTOR inhibitors use (control group) were further analyzed, and the results were not changed. In addition, the dose exposure analysis showed a consistent class effect. mTOR inhibitors dose exposure did not change the risk of cancer development (Table 4), or all-cause mortality (Table 5) when compared to that in non-users group. No difference was found in either the “sirolimus only” or “everolimus only” group analysis.

**Table 4:** Exposure of mTOR inhibitors and risk of cancer development.

	Events/patients	IR (per 1,000 person-years)	Total person-months	Adjusted HR* (95% CI)	*p*-value
Non-mTOR inhibitor users	598/3,541	23.7	302,160	1.0	
Sirolimus or everolimus					
Cumulative**					
≤224 DDDs	31/312	16.8	22,117	0.79(0.55–1.14)	0.204
225–500.7 DDDs	28/298	14.1	23,761	0.71(0.48–1.04)	0.079
≥500.8 DDDs	48/305	21.1	27,256	1.04(0.76–1.40)	0.826
Average daily					
≤0.613 DDDs	30/303	17.0	21,178	0.78(0.54–1.13)	0.193
0.614–1.37 DDDs	29/306	14.2	24,592	0.70(0.48–1.02)	0.066
≥1.38 DDDs	48/306	21.0	27,364	1.03(0.76–1.40)	0.830
Sirolimus (only)					
Cumulative					
≤3.3 DDDs	5/17	53.1	1,129	2.56(1.05–6.21)	0.038
3.4–9 DDDs	0/18	–	1,666	–	–
≥10 DDDs	4/18	37.7	1,273	1.59(0.59–4.28)	0.359
Average daily					
≤0.0091 DDDs	5/17	53.1	1,129	2.56(1.05–6.21)	0.038
0.0092–0.026 DDDs	0/18	–	1,666	–	–
≥0.027 DDDs	4/18	37.7	1,273	1.59(0.59–4.28)	0.359
Everolimus (only)					
Cumulative					
≤4.7 DDDs	0/18	–	694	–	–
4.8–13 DDDs	1/19	16.2	741	0.93(0.13–6.69)	0.945
≥14 DDDs	0/17	–	678	–	–
Average daily					
≤0.013 DDDs	0/18	–	694	–	–
0.014–0.035 DDDs	1/17	16.2	741	0.89(0.12–6.33)	0.903
≥0.036 DDDs	0/19	–	678	–	–

**Notes:**

* Adjusted for age, gender, dialysis vintage, transplant within/overseas, DM, HTN, CHF, ACEi/ARB, Beta-blocker, Aspirin, NSAID, Statins, Cyclosporin, Tacrolimus, Mycophenolic acid, ATG.

** Cumulative defined daily dose (cDDD): summing the total DDD during the study period; Average daily dose: cDDD divided by cumulative prescription days. DDD Sirolimus three mg; Everolimus 1.5 mg.

**Table 5:** Exposure of mTOR inhibitors and risk of all-cause mortality.

	Events/patients	IR (per 1,000 person-years)	Total person-months	Adjusted HR* (95% CI)	*p*-value
Non-mTOR inhibitor users	432/3,541	15.6	332,228	1.0	
Sirolimus or Everolimus					
Cumulative**					
≤224 DDDs	28/312	14.2	23,693	0.96(0.65–1.42)	0.832
225–500.7 DDDs	35/298	16.7	25,174	1.33(0.93–1.89)	0.114
≥500.8 DDDs	35/305	14.3	29,291	1.11(0.78–1.58)	0.574
Average daily					
≤0.61 DDDs	27/303	19.5	22,739	0.95(0.64–1.41)	0.798
0.62–1.37 DDDs	36/306	16.6	26,020	1.33(0.94–1.88)	0.108
≥1.38 DDDs	35/306	14.3	29,399	1.11(0.78–1.58)	0.577
Sirolimus (only)					
Cumulative					
≤3.3 DDDs	3/17	27.5	1,311	2.34(0.75–7.33)	0.146
3.4–9 DDDs	1/18	7.2	1,666	0.39(0.05–2.80)	0.348
≥10 DDDs	4/18	31.6	1,517	2.07(0.77–5.59)	0.152
Average daily					
≤0.0091 DDDs	3/17	27.5	1,311	2.34(0.75–7.33)	0.146
0.0092–0.026 DDDs	1/18	7.2	1,666	0.39(0.05–2.80)	0.348
≥0.027 DDDs	4/18	31.6	1,517	2.07(0.77–5.59)	0.152
Everolimus (only)					
Cumulative					
≤4.7 DDDs	1/18	17.3	694	2.81(0.38–19.47)	0.305
4.8–13 DDDs	0/19	–	752	–	–
≥14 DDDs	1/17	17.7	678	2.71(0.38–19.75)	0.322
Average daily					
≤0.013 DDDs	1/18	17.3	694	2.84(0.39–20.42)	0.301
0.014–0.035 DDDs	0/17	–	752	–	–
≥0.036 DDDs	1/19	17.7	678	2.75(0.38–19.75)	0.315

**Notes:**

IR: incidence rate, per 1,000 person-years.

* Adjusted for age, gender, dialysis vintage, transplant within/overseas, DM, HTN, CHF, ACEi/ARB, Beta-blocker, Aspirin, NSAID, Statins, Cyclosporin, Tacrolimus, Mycophenolic acid, ATG.

** Cumulative defined daily dose (cDDD): summing the total DDD during the study period; Average daily dose: cDDD divided by cumulative prescription days. DDD Sirolimus three mg; Everolimus 1.5 mg.

## Discussion

Prolonged survival and the use of cumulative immunosuppressive doses lead to the development of cancer after kidney transplantation. Cancer-related morbidity and mortality are one of the leading causes of death after kidney transplantation (Russ, 2013). In this study, we found that treatment with mTOR inhibitors did not reduce the risk of *de novo* cancer development in kidney transplantation patients, a result that contradicts that of previous studies (Ekberg et al., 2007; Kasiske et al., 2004). Most of the beneficial effects of mTOR inhibitors on cancer were from the reduction of skin cancer (Euvrard et al., 2012; Knoll et al., 2014; Opelz et al., 2016). However, the development of skin cancer after kidney transplantation is very limited in Taiwan, as reported both by our study and by a previous publication (Li et al., 2012). The specific anti-cancer effect of mTOR inhibitors on skin cancer could not be explained in our study. The mechanisms responsible for this anti-cancer effect have not been elucidated yet. One potential explanation could be based on the immune regulation of mTOR inhibitors. mTOR inhibitors do not only block IL-2 proliferation but also alter T cell proliferation through promotion of regulatory T cell (Treg) development (Zeiser et al., 2008). The specific role of Treg in different types of cancer may differ (Mougiakakos et al., 2010) and therefore, the effect of mTOR inhibitors on different cancer types may differ too. Another explanation could be based on viral infections that are highly associated with post-transplantation skin cancer (Nindl & Rosl, 2008). mTOR inhibitors have anti-viral effects (Brennan et al., 2013) and this may also account for their specific anti-skin cancer effect (Geissler, 2015).

Our study showed no significant differences in all-cause mortality between the groups. Our cohort presented a long follow-up period, with a median time of 7.8 years. To further understand if any specific cause of mortality contributes to the outcome, we performed a subgroup analysis. No increased risk of any specific cause of mortality was shown. Previous studies showed heterogeneous results when analyzing mTOR inhibitors and their effects on the risk of all-cause mortality. Cortazar et al. (2012) reported that patients who used mTOR inhibitors had a higher risk of mortality in a 3-year follow-up. Isakova et al. (2013) showed that the primary use of mTOR inhibitors carried a worse outcome and increased the risk of death up to 8 years after kidney transplantation. In addition, a meta-analysis (Knoll et al., 2014) reported that sirolimus was associated with an increased risk of death in patients with kidney transplantation. Several factors may explain this increased risk of death. mTOR inhibitors are known to have an adverse effect on dyslipidemia, glucose intolerance, and high blood pressure; thus they may increase the risk of CV events (Zaza et al., 2013). Moreover, mTOR inhibitors may cause proteinuria (Letavernier & Legendre, 2008) which underlies the endothelial cell dysfunction, and vascular comorbidities. In addition, mTOR inhibitors users may have a higher risk of rejection. Episodes of rejection may expose patients to more immunosuppressives and increase the risk of infection; subsequently increasing the risk of death (Isakova et al., 2013). However, neither CV-related nor infection-related mortality were increased in our subgroup analysis.

Nevertheless, other studies showed that the risk of mortality in mTOR inhibitor users was not increased. A meta-analysis showed no significant difference in mortality between mTOR inhibitors users and non-users (Webster et al., 2006). Another meta-analysis also found that in an everolimus-based regimen, the risk of mortality was not increased (Su et al., 2014). There are several factors that may explain the lack of adverse effects on survival. Mainly, the anti-proliferative effect of mTOR inhibitors may have benefits on atherosclerosis and reduces the risk of CV disease (Morice et al., 2002). In addition, mTOR inhibitors have been reported to improve vasculopathy in cardiac transplantation patients (Mancini et al., 2003; Sinha et al., 2008). This may account for the reduced risk of a composite of CV and renal outcome in the propensity score matching model. In addition, cytomegalovirus infection, a deliberating issue in post-kidney transplantation, is decreased in mTOR inhibitors users (Lim et al., 2014; Nashan et al., 2012). Further studies are needed to elucidate the effects of mTOR inhibitors.

The conversion from CNIs to mTOR inhibitors induces protection against cancer. Previous studies have shown reduced risk of cancer development after conversion of CNIs into mTOR inhibitors (Alberu et al., 2011; Campistol et al., 2006; Lebranchu et al., 2011; Lim et al., 2014). Our study did not show any effect either by conversion or by *de novo* use of mTOR inhibitors. The exact mechanisms through which cancer risk decreases in the conversion group are not known (Halleck & Budde, 2012). The beneficial effects of conversion on cancer development may have indication bias due to the selection of patients (Cortazar et al., 2012). Our stratified analysis showed that mTOR inhibitors were associated with the protective effect in cyclosporine users and statin non-users subgroups. We speculated patients in the subgroup of cyclosporine users might discontinue it later in the study period, and therefore presented protective effect. Statins are known to have anti-cancer effect by anti-angiogenic and anti-inflammatory pathways (Chan, Oza & Siu, 2003; Dulak & Jozkowicz, 2005). We inferred the beneficial effects hidden by the statins use, which may explain the result in this subgroup. Regarding all-cause mortality, the stratified analysis found mTOR inhibitors were not associated with risk alterations, except for that in subgroups of tacrolimus users and MPA non-users. The combination of tacrolimus and mTOR inhibitors was associated with increased risk of metabolic syndromes from 11.0% to 38.1%, (Verges, 2018) which may contribute to the risk of CV disease and allograft dysfunction. Without MPA, higher rejection and graft failure rates may be observed (Shapiro et al., 1999). Though the above findings may have some pathophysiological background, the specific cause of mortality showed no significant findings. In addition, the subgroup analysis after Bonferroni correction revealed negative findings. Further investigations are needed in this subject.

The strength of our study lies in the use of a nation-wide database that may better represent a real-world setting. It is a comprehensive, reliable database and consists of a long-term follow up. We have the complete operation codes and these patients had thorough records that must conform to Taiwan’s health insurance system when uploaded. Second, our study investigated the specific cancer and the specific cause of mortality by linking the database records to the medical certification of death profiles; which is comparably valuable to previous reports (Budde et al., 2015; Isakova et al., 2013; Lim et al., 2014). Third, we studied the impact of overall dose exposure of mTOR inhibitors on the outcomes. To our knowledge, there are no studies testing the dosage effect of mTOR inhibitors on cancer. Previous studies evaluated the exposure to mTOR inhibitors at enrollment only and then compared it to subsequent outcomes (Cortazar et al., 2012; Opelz et al., 2016). Drug exposure and its effects might be most appropriately represented by its cumulative dose. To investigate its potential effects, we calculated the cumulative dose of mTOR inhibitors during the whole study period and determined the effect of dose exposure on the outcome. We analyzed the doses of each drug (sirolimus and everolimus) via DDD and determined the class effect of mTOR inhibitors. Further, both mTOR inhibitors were investigated separately. Our results were consistent and similar to the primary outcome. Neither high- nor low-dose of mTOR inhibitors was associated with a reduced risk of cancer or of all-cause mortality. All statistical models used showed similar results.

However, our study also has some limitations. First, we divided patients into two groups according to a 180-day cumulative use of mTOR inhibitors, which may underestimate the effect of mTOR inhibitors. To reduce the misclassification effects, we used different cutoffs for mTOR inhibitors usage and analyzed the patients without any mTOR inhibitors as a control group. The results were not changed. These analyses alleviated the potential misclassification group effect on the outcomes. Second, the NHIRD is an administrative database and several covariates were not identified. These include socioeconomic status, donor type, and use of induction therapy. Induction therapy is mostly not included in Taiwan’s health insurance system. Data on rejection episodes were not available, which may affect the graft and patient survival. However, Taiwan’s health insurance provides complete coverage and equal opportunities for all the transplant recipients. It also subsidizes all the co-payments to reduce financial barriers. Therefore, the difference of medical care in these patients should be minimal. Third, although our sample size could not discriminate between the outcome differences, we found that the trend is toward a protective effect against cancer development, and a deleterious effect on mortality, which is consistent with previous literature. Based on the HR of our result, we calculated that an enrollment of 17,636 patients would have a power of 80% to detect differences in cancer development. A longer follow-up duration may be warranted to detect differences.

## Conclusions

We could not find the association of mTOR inhibitor use and risk of *de novo* cancer development or all-cause mortality among patients in Taiwan. The analysis of specific cancer subtypes or specific causes of mortality did not show any significant differences between the groups. Cumulative exposure to mTOR inhibitors showed similar results.

## Supplemental Information

10.7717/peerj.5864/supp-1 Supplemental Information 1 Table S1. Association of mTOR inhibitors and cancer development in propensity score matching model

10.7717/peerj.5864/supp-2 Supplemental Information 2 Table S2. Association of mTOR inhibitors and specific cause of mortality in propensity score matching model

10.7717/peerj.5864/supp-3 Supplemental Information 3 Table S3. Subgroup analysis of cancer risk in mTOR inhibitors user after Bonferroni correction

10.7717/peerj.5864/supp-4 Supplemental Information 4 Table S4. Subgroup analysis of all-cause mortality risk in mTOR inhibitors user after Bonferroni correction

## Additional Information and Declarations

### Data Availability

The following information was supplied regarding data availability: The raw data are provided in the Supplemental Files.
