# Supplementary material for: Cancer and mTOR inhibitors in kidney transplantation recipients
Source: PeerJ. 2018 Nov 8;6:e5864. doi: 10.7717/peerj.5864 (PMC6237112; doi:10.7717/peerj.5864)
Supplement: Supplemental Information 1 [file peerj-06-5864-s001.docx]

**Supplemental file**

| Supplementary Table 1. Association of mTOR inhibitors and cancer development in propensity score matching model | | |
| --- | --- | --- |
| Outcome | Propensity score matching model | P |
|  | HR (95% CI) |  |
| Cancer | 0.92 (0.81-1.08) | 0.33 |
| Kidney | 0.81 (0.54-1.20) | 0.30 |
| Bladder | 0.83 (0.59-1.15) | 0.26 |
| Lung | 0.64 (0.24-1.74) | 0.38 |
| Liver | 0.81 (0.49-1.34) | 0.41 |
| Colorectal | 1.41 (0.64-3.12) | 0.39 |
| Breast | 0.25 (0.06-1.13) | 0.07 |
| Hematological | 1.07 (0.54-2.12) | 0.86 |
| Skin | 0.71 (0.10-4.87) | 0.73 |
| Prostate | 1.08 (0.43-2.69) | 0.87 |
| Others | 0.95 (0.73-1.22) | 0.67 |

CI: confidence interval; HR: Hazards ratio
