# Supplementary material for: Cancer and mTOR inhibitors in kidney transplantation recipients
Source: PeerJ. 2018 Nov 8;6:e5864. doi: 10.7717/peerj.5864 (PMC6237112; doi:10.7717/peerj.5864)
Supplement: Supplemental Information 2 [file peerj-06-5864-s002.docx]

**Supplemental file**

| Supplementary Table 2. Association of mTOR inhibitors and specific cause of mortality in propensity score matching model | | |
| --- | --- | --- |
| Outcome | Propensity score matching model | P |
|  | HR (95% CI) |  |
| All-cause mortality | 0.96 (0.81-1.13) | 0.60 |
| Malignancy | 1.39 (1.01-1.90) | 0.04 |
| CV | 0.66 (0.38-1.14) | 0.13 |
| CV/Renal | 0.73 (0.55-0.97) | 0.03 |
| Infectious | 1.06 (0.55-2.02) | 0.87 |
| Accident | 0.68 (0.11-4.15) | 0.67 |
| Others | 0.93 (0.69-1.27) | 0.66 |

CV: cardiovascular; DM: diabetes mellitus; HR: Hazards ratio; CI: confidence

interval
