# Supplementary material for: Cancer and mTOR inhibitors in kidney transplantation recipients
Source: PeerJ. 2018 Nov 8;6:e5864. doi: 10.7717/peerj.5864 (PMC6237112; doi:10.7717/peerj.5864)
Supplement: Supplemental Information 3 [file peerj-06-5864-s003.docx]

| Supplementary Table 3. Subgroup analysis of cancer risk in mTOR inhibitors user after Bonferroni correction | | | | |
| --- | --- | --- | --- | --- |
| Subgroup |  | HR (95% CI) | P value | Bonferroni  P value |
| Statin | user | 0.92 (0.57-1.47) | 0.71 | >0.99 |
|  | nonuser | 0.71 (0.47-1.07) | 0.10 | >0.99 |
| Cyclosporin | user | 0.66 (0.41-1.06) | 0.09 | >0.99 |
|  | nonuser | 0.91 (0.61-1.37) | 0.65 | >0.99 |
