# Supplementary material for: Cancer and mTOR inhibitors in kidney transplantation recipients
Source: PeerJ. 2018 Nov 8;6:e5864. doi: 10.7717/peerj.5864 (PMC6237112; doi:10.7717/peerj.5864)
Supplement: Supplemental Information 4 [file peerj-06-5864-s004.docx]

| Supplementary Table 4. Subgroup analysis of all-cause mortality risk in mTOR inhibitors user after Bonferroni correction | | | | |
| --- | --- | --- | --- | --- |
| Subgroup |  | HR (95% CI) | P value | Bonferroni  P value |
| Tacrolimus | user | 1.27 (0.87-1.86) | 0.22 | >0.99 |
|  | nonuser | 0.54 (0.23-1.24) | 0.14 | >0.99 |
| Mycophenolic acid | user | 0.97 (0.67-1.42) | 0.88 | >0.99 |
|  | nonuser | 2.12 (0.97-4.64) | 0.06 | >0.99 |
